# Identification of Primary Congenital Hypothyroidism Based on Two Newborn Screens — Utah, 2010–2016

**DOI:** 10.15585/mmwr.mm6728a4

**Published:** 2018-07-20

**Authors:** David E. Jones, Kim Hart, Stuart K. Shapira, Mary Murray, Robyn Atkinson-Dunn, Andreas Rohrwasser

**Affiliations:** ^1^Utah Public Health Laboratory, Salt Lake City, Utah; ^2^Office of the Director, National Center on Birth Defects and Developmental Disabilities, CDC; ^3^University of Utah, Salt Lake City, Utah.

Newborn screening for primary congenital hypothyroidism is part of the U.S. Recommended Uniform Screening Panel (*1*,*2*). Untreated congenital hypothyroidism can result in cognitive impairment and growth complications (decreased height/length). Initial newborn screening for congenital hypothyroidism is typically performed 24–48 hours after birth. Fourteen states, including Utah, perform a routine second screen at approximately 2 weeks of age.* During 2010–2016, a total of 359,432 infants in Utah were screened for congenital hypothyroidism, and 130 cases were diagnosed; among these, 98 had an abnormal first screen, and 25 had an abnormal second screen (seven infants were excluded because of missing data). A retrospective examination of Utah’s screening data indicated that 20% of congenital hypothyroidism cases could not have been efficiently identified by a single screen alone. This study highlights the utility of a two-screen process and demonstrates that differential cutoff values for the first and second screens could optimize both screening sensitivity and specificity.

Congenital hypothyroidism is a pediatric disorder with an observed prevalence in the United States of one in 2,000–4,000 live births (*3*) and a prevalence in Utah of one in 2,800. Early detection and initiation of treatment within the first 30 days of life substantially reduce the risk for permanent cognitive impairment (*4*). Newborn screening for congenital hypothyroidism in Utah is accomplished by measuring thyroid-stimulating hormone (TSH) from dried whole blood spots collected on a newborn screening card by heel stick. The first specimen (first screen) is collected within 24–48 hours of life; the second specimen (second screen) is collected during 7–28 days of life. All infants receive two screens, even if the first screen is positive. In Utah, during this study period, any TSH value ≥40 *μ*IU/mL is considered abnormal for both the first and second screens; elevated screening results are followed by diagnostic testing. From the perspective of this study, a confirmed case is defined as an abnormal newborn screen (elevated TSH) as well as a clinical diagnosis of congenital hypothyroidism.

Among 130 confirmed cases of congenital hypothyroidism identified in Utah during 2010–2016, 123 cases with two screens were analyzed, including 98 cases identified by the first screen and 25 cases identified by the second screen; seven of the 130 cases were excluded because only one test result was available. Infants with confirmed congenital hypothyroidism were stratified into two groups: those with an abnormal first screen (group 1) and those with a normal first screen but an abnormal second screen (group 2). Mean TSH concentrations for both the first and second screens were computed and compared for both groups. Student’s t-tests were performed to test for significant differences in TSH concentration as a function of group. A retrospective cutoff analysis was performed to determine whether all group 2 cases (those identified only on the second screen) could be identified by a single screen. This retrospective cutoff analysis involved analyzing the number of false positives and false negatives as a function of adjusting the first screen cutoff value (range = 5–40 *μ*IU/mL). To ensure infants with persistent but only marginally elevated TSH concentrations are identified, the numbers of screened infants with a TSH concentration 20 *μ*IU/mL–40 *μ*IU/mL on the first or second screen during 2010–2016 were determined and compared.

Mean TSH concentrations varied as a function of group and by screen number (first vs. second) (Table). The highest TSH concentrations were observed in group 1 infants on the first screen. Among group 2 infants (those with cases diagnosed on the second screen), TSH concentrations were lower on the first screen, with all infants having concentrations below the cutoff value of 40 *μ*IU/mL (range = 5.3–39.8 *μ*IU/mL) resulting in normal first screen designation, but with TSH concentrations above the cutoff level on the second screen (Figure 1). Compared with all infants screened, TSH levels in group 1 and group 2 infants were significantly elevated on both the first and second screens (Table).

**TABLE:** Mean thyroid-stimulating hormone (TSH) levels on first and second congenital hypothyroidism screening tests among 123 infants with congenital hypothyroidism and comparison within and between groups — Utah, 2010–2016

Population	First screen*	Second screen*	P-value
Group 1 (n = 98)^†^ (mean TSH [*μ*IU/mL])	397.3	215.8	<0.001
Group 2 (n = 25)^§^ (mean TSH [*μ*IU/mL])	23.9	107.8	0.002
All infants screened (n = 359,432) (mean TSH [*μ*IU/mL])	10.7	3.9	<0.001
**Comparison between groups**			
Group 1 versus group 2 (p-value)	<0.001	0.022	NA
Group 1 versus all infants screened (p-value)	<0.001	<0.001	NA
Group 2 versus all infants screened (p-value)	<0.001	<0.001	NA

**Abbreviations:** IU = international unit; NA = not applicable.

* During the time frame of this study the normal TSH concentration level was <40 *μ*IU/mL.

^†^ Confirmed congenital hypothyroidism with abnormal TSH levels on first screen.

^§^ Confirmed congenital hypothyroidism with normal TSH level on first screen and abnormal TSH level on second screen.

**FIGURE 1:**
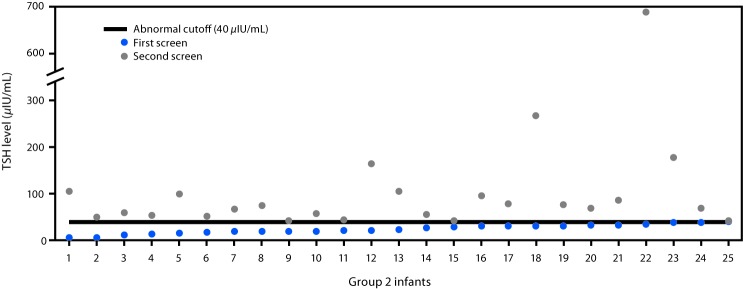
Thyroid-stimulating hormone (TSH) levels among 25 infants with congenital hypothyroidism who had a normal first screen and an abnormal second screen (group 2 infants) — Utah, 2010–2016

When concurrently examining the number of cases with false-positive and false-negative results (missed cases) as a function of TSH concentration cutoff, an inverse relationship was observed (Figure 2). A moderate cutoff adjustment from 40 *μ*IU/mL to 20 *μ*IU/mL would have resulted in approximately 27,600 false-positive and 11 missed cases. To ensure that all group 2 cases were detected through a single screen, a TSH cutoff value of 5 *μ*IU/mL would have been necessary, which would have resulted in approximately 282,850 false-positive cases or approximately 79% of the screened population.

**FIGURE 2:**
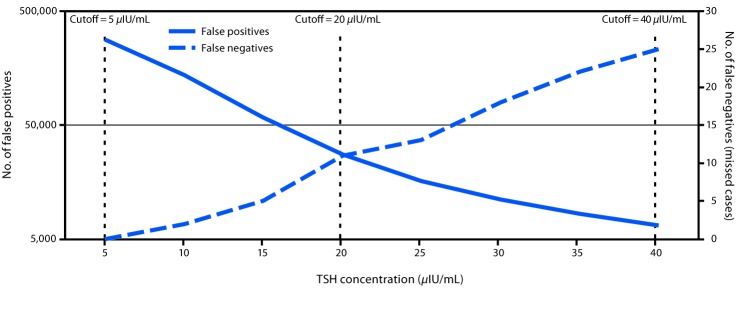
Retrospective comparison of number of false positives and false negatives on the first newborn screen using different thyroid-stimulating hormone (TSH) cutoff values — Utah, 2010–2016

## Discussion

The goal of newborn screening is to not miss cases, while avoiding overwhelming the health care system with false-positive screens requiring unnecessary follow-up and diagnostic testing. In Utah, a two-screen program supports this goal. During 2010–2016, approximately 20% of all confirmed congenital hypothyroidism cases were identified through the second screen. A retrospective analysis that examined lowering the abnormal TSH cutoff value indicated that cases identified only on the second screen could not have been identified through a single screen. Even with a moderate cutoff value adjustment from 40 *μ*IU/mL to 20 *μ*IU/mL, 44% (11/25) of cases would have been missed by a single screen.

Two-screen programs with a similar screening cutoff for both screens risk missing infants with marginally elevated TSH concentrations, who need to be treated (*5*,*6*). Congenital hypothyroidism detection is directly related to cutoff values, which vary among newborn screening programs (*7*). The 2016 CDC Newborn Screening Quality Assurance Program Annual Summary Report indicated that the mean and mode TSH cutoff values for U.S. newborn screening laboratories were 30.6 *μ*IU/mL and 20 *μ*IU/mL, respectively.^†^ In light of higher TSH concentration observed during the first screen period, but lower TSH concentrations during the 7–28 day period, higher TSH cutoff values for the first period are advisable. This is consistent with the observed lower TSH concentrations of both groups (i.e., those with abnormal first or second screens) for the second screen.

Among infants screened during 2010–2016, TSH concentrations of 20 *μ*IU/mL–40 *μ*IU/mL were found for approximately 21,000 infants on the first screen with 85 remaining elevated on the second screen. A lower screening cutoff for the first screen would obligate reporting many infants with positive screens to primary care providers, potentially resulting in unnecessary additional testing as well as unnecessary stress for families and providers.

This data-driven analysis, with the goal of optimizing screening sensitivity and specificity, identified two potential workflow adaptations that would allow identification of all group 2 cases together with infants with marginally elevated TSH concentrations. For the first approach, infants with TSH concentrations 20 *μ*IU/ml–40 *μ*IU/ml on the first screen could be characterized as “borderline.” These cases would be reported as “abnormal” only if the TSH concentration would exceed 20 *μ*IU/ml during the second screen as well. A second approach would use a tiered cutoff, with a TSH concentration cutoff of 40 *μ*IU/mL on the first screen and 20 *μ*IU/mL on the second screen to identify these cases.

Retrospective analysis of Utah data indicated that a two-screen approach with a cutoff of 40 *μ*IU/mL on the first screen and 20 *μ*IU/mL on the second screen, followed by referral of infants with TSH values above each screen’s cutoff for diagnostic evaluation, would result in approximately 1,000 infants per year, or approximately 2% of Utah’s annual screening volume, requiring follow-up and diagnostic testing. This reflects a modest increase over the current workload of approximately 950 infants requiring follow-up and diagnostic testing for congenital hypothyroidism each year. This approach would detect infants with persistent marginally elevated TSH concentration (20 *μ*IU/mL–40 *μ*IU/mL), identify both group 1 and group 2 congenital hypothyroidism cases, and improve the sensitivity and specificity of congenital hypothyroidism screening.

The findings in this report are subject to at least one limitation based on the small size of the sample, which included only 130 confirmed cases of congenital hypothyroidism among a sample of 359,432 infants. Although the observed differential progression of TSH elevation might suggest heterogeneous disease mechanisms for congenital hypothyroidism, further elucidation of underlying differential pathophysiology and molecular mechanisms would require replication in larger cohorts. Such efforts could be further complicated by genetic and population heterogeneity. However, similar findings have been observed by other two-screen programs regarding the importance of a second screen for identifying cases of congenital hypothyroidism (*7*)

Analysis of 7 years of newborn screening data for congenital hypothyroidism in Utah demonstrated the value and benefits of a two-screen program. Identifying all congenital hypothyroidism cases through a single screen would have required a cutoff of 5 *μ*IU/mL and would have required diagnostic testing for 79% of the population. Cases of congenital hypothyroidism identified through the first screen had significantly higher mean TSH concentrations compared with cases identified through the second screen. These significant differences suggest that applying the same screening cutoff limits to both first and second screens might result in missed cases of congenital hypothyroidism with marginally elevated TSH levels. Applying the same cutoffs might also miss cases because of the known physiologic TSH concentration changes in relation to age of the infant and specimen collection (*6*,*8*). The suggested workflow adaptations could help ensure that no cases of congenital hypothyroidism are missed.

**Summary**

**What is already known about this topic?**

Screening for congenital hypothyroidism is conducted by all newborn screening programs in the United States.

**What is added by this report?**

Retrospective analysis of 7 years of Utah newborn screening data found that 20% of congenital hypothyroidism cases were in infants who had normal thyroid-stimulating hormone (TSH) concentrations on the first screen but elevated TSH concentrations on the second screen. One screen alone could not have identified all of these cases.

**What are the implications for public health practice?**

This study underscores the utility and power of a two-screen approach in identifying congenital hypothyroidism cases with normal TSH concentrations on the first screen but elevated TSH concentrations on the second screen. A two-screen approach also limits the number of false positive cases.

## Footnotes

* https://www.newsteps.org/resources/number-required-newborn-screens-regional-genetics-networks-and-number-annual-births.

† https://www.cdc.gov/labstandards/pdf/nsqap/nsqap_summaryreport_2016.pdf.
